# Serological prevalence and persistence of high-risk human papillomavirus infection among women in Santiago, Chile

**DOI:** 10.1186/1471-2334-14-361

**Published:** 2014-07-03

**Authors:** Felipe A Castro, Angelica Dominguez, Klaus Puschel, Vanessa Van De Wyngard, Peter JF Snijders, Silvia Franceschi, Michael Pawlita, Catterina Ferreccio

**Affiliations:** 1 Division of Cancer Epidemiology and Genetics, National Cancer Institute, National Institutes of Health, 6120 Executive Blvd, Rockville, MD 20852, USA; 2 Facultad de Medicina, Pontificia Universidad Católica de Chile, Marcoleta 434, Santiago 8330073, Chile; 3 Department of Pathology, VU University Medical Center, De Boelelaan 1117, 1081 HV Amsterdam, The Netherlands; 4 International Agency for Research on Cancer, 150 cours Albert Thomas, 69372 Lyon cedex 08, France; 5 Division of Genome Modifications and Carcinogenesis, German Cancer Research Center (DKFZ), Im Neuenheimer Feld 280, Heidelberg D-69120, Germany; 6 Centro FONDAP/Advanced Center for Chronic Diseases (ACCDiS), Marcoleta 434, Santiago 8330073, Chile; 7 Division of Clinical Epidemiology and Aging Research, German Cancer Research Center (DKFZ), Im Neuenheimer Feld 581, Heidelberg 69120, Germany

**Keywords:** Human papillomavirus, Seropersistence, Cohort, Serology, Antibodies, Natural history

## Abstract

**Background:**

Human papillomavirus (HPV) serology is a main factor for designing vaccination programs and surveillance strategies; nevertheless, there are few reports of HPV seroprevalence in the general population, especially in Latin America. This study aimed to describe high-risk HPV serological prevalence, persistence, and association with concurrent cervical infection, in Chilean women.

**Methods:**

1021 women from the general population, aged 15–85 years, were studied in 2001 of whom 600 were reexamined in 2006. The assessments at both time points included cervical HPV DNA testing, HPV antibody testing, cervical cytology and a sociodemographic/behavioral questionnaire. HPV DNA and antibodies against L1 protein of types 16, 18, 31, 33, 35, 45, 52, and 58 were assessed by reverse line blot and multiplex serology, respectively.

**Results:**

Seropositivity was high at both baseline (43.2%) and follow-up (50.2%) and increased with age (p < 0.001); corresponding DNA prevalences were 6.7% and 8.7%. DNA and seroprevalence were associated at baseline (p = 0.01 for any HPV). Early age at first sexual intercourse and having had two or more sexual partners were independently associated with seropositivity. Most (82.0%) initially seropositive women remained seropositive at follow-up; 21.6% of initially seronegative women seroconverted, reaching 17.5% among women older than 60 years of age. ASCUS or worse cytology was correlated with HPV DNA positivity but not with HPV seropositivity.

**Conclusion:**

HPV seroprevalence studies are a useful tool for learning about the dynamics of HPV infection in a community. This study contributes to understanding the natural history of HPV infection and provides a baseline assessment before the incorporation of HPV vaccination into a national program.

## Background

Chile is a Latin American country where screening programs for cervical cancer have been effective in decreasing the burden of this malignancy [1]. Nevertheless, 1478 women are diagnosed with cervical cancer and approximately 600 die from the disease every year in Chile. Age-standardized cervical cancer mortality rate of 5.7/100,000 [2] in Chile is still substantially higher than the rates reported in the majority of developed countries [3,4]. Women with low socio-economic status are most affected by this disease [5].

Human papillomavirus (HPV) is the most common sexually transmitted infection in the world and persistent infection with high-risk HPV (HR-HPV) types is a necessary cause of cervical cancer [6]. A population-based survey of adult Chilean women reported an overall prevalence of HR-HPV genital infection (any oncogenic type) of 15%, with the highest prevalence observed in women under 25 years old [7]. A subsequent study confirmed that, among women in Santiago, Chile, cervical infection with HR-HPV peaks at young ages (< 20 years old) and then steadily decreases to stabilize around age 40 years, and increases again after age 60 years [8].

While HPV DNA informs about current cervical infection, incident or persistent, it is not a marker for cumulative exposure to the virus [9]. Cumulative exposure is best determined by measurement of serum IgG antibodies against HPV, which are considered the footprints left by infections that occurred during the lifetime of the individuals because they persist after DNA becomes undetectable [10,11]. Although HPV serology is an imperfect measure of past exposure since almost half of DNA-positive women are seronegative [12,13], today it is considered a marker of cumulative HPV infection [14]. Seroprevalence studies have been useful in understanding the natural history of HPV infection and in evaluating HPV exposure in the population to identify target groups for HPV vaccination programs. Also, the recommendation of HPV immunization in girls is based on “immunological bridging”, that is, the demonstration of similar or higher antibody levels in girls as in women in whom clinical efficacy against cervical carcinoma in situ was shown [15]; and the duration of vaccine protection and the need of a booster dose will be based partly on serological surveillance of HPV antibodies. In particular, multiplex assays for detection of antibodies against several HPV types have an interesting potential in epidemiologic studies; evaluations of such methods in prospective cohorts provide additional information about the utility of seroassays and about the serologic response to HPV infection in specific populations.

As part of a population-based study of the natural history of HPV infection in women from Santiago, Chile [8,16], we evaluated here seroprevalence and seropersistence and their correlates for the eight most common HR-HPV types (16, 18, 31, 33, 35, 45, 52 and 58) using a Luminex-based serology assay.

## Methods

### Study population

The present analysis was performed on a random sample of 1393 Chilean women from the general population of a low socio-economic area of Santiago, Chile. A detailed description of the study design, enrollment, data collection procedures and results of HPV DNA infection has been published previously [16]. Briefly, eligible women were those aged 15 years and older, who were sexually active, not pregnant, covered by the national health insurance system and who had no medical impediment to participate. Women were examined at enrollment in 2001 and follow-up in 2006; assessment at both time points included Pap cytology, cervical HPV DNA testing, HPV antibody testing and a sociodemographic and behavioral questionnaire [8]. A total of 1221 (87.6%) agreed to participate at baseline and 689 (56.4%) of them participated at follow-up. Causes of non-participation in follow-up have been reported elsewhere [8]. The analysis of seroprevalence was restricted to 1021 women who had complete serologic data at baseline, and the analysis of seropersistence to 600 women who had serological results for both baseline and follow-up visits. Participants who completed follow-up were slightly different from those who did not: they were more likely to have a low education level, to be single and to have fewer children and fewer sexual partners at baseline; however, they had similar any HPV DNA prevalence (5.9% and 7.5% respectively), any HPV seroprevalence (44.5% and 41.6% respectively), proportion of seropositive women with multiple HPV types (42.3% and 48.6% respectively) and prevalence of ASCUS or worse (ASCUS+) cytology (3.7% both groups) (Additional file 1).

All participants gave written informed consent. The study protocol was approved by the ethical committees of the Ministry of Health’s South-Eastern Health Service and of the International Agency for Research on Cancer.

### Sample collection

Collection of cervical samples for HPV DNA testing has been described previously [8,16]. Briefly, women attended a health center where a midwife collected exfoliated cervical cells using an Ayre spatula which was then placed in a tube with PBS; additionally, a spatula and cytobrush used for a Pap smear obtained at the same visit were washed in this tube. The samples were centrifuged at 3,000 × g for 10 minutes and the resulting pellet was diluted in saline solution and stored at -30°C until sent for analysis. A 10 ml blood sample was collected from all consenting participants. Blood samples were centrifuged at 1500 × g for 10 min and serum was divided into different aliquots. Serum samples were stored first at -20°C for no longer than one week and then at -70°C until sent for analysis. A total of 36 serum samples (18 collected in 2001 and 18 in 2006) were excluded due to insufficient sample material.

### HPV DNA testing

The method used for HPV DNA testing has been described in detail elsewhere [8,16]. Briefly, cervical samples were analyzed at the Department of Pathology of the VU University Medical Center in Amsterdam, The Netherlands. Genotyping was performed using a GP5+/6+ primer-mediated PCR and enzyme immune assay. Reverse line blot genotyping was used to identify HR-HPV types 16, 18, 31, 33, 35, 45, 52 and 58.

### HPV multiplex serology

Serum samples were analyzed at the German Cancer Research Center (DKFZ), Heidelberg, Germany. The presence of IgG antibodies against the major capsid protein L1 of HR-HPV types 16, 18, 31, 33, 35, 45, 52 and 58 was assessed using multiplex serology based on glutathione S-transferase fusion proteins, which has been broadly used in epidemiological studies [14]. The included HPV types were selected because they are the most frequent types in cervical cancer worldwide [17]; in Chile, they are present in 98.3% of cervical cancers [18]. The assay was previously described in detail [19,20]. Briefly, fluorescence-coded bead sets (3000 beads per set per well) carrying different HPV antigens were mixed and incubated with serum diluted to 1:100 in 96-well plates. The plates were incubated on a shaker in the dark at room temperature for one hour. The beads were washed three times with 100 ml casein buffer on a vacuum manifold. Secondary biotinylated antibody and the fluorescent reporter conjugate streptavidin-R-phycoerythrin were added and incubated for one hour and 30 minutes, respectively, with washing steps in between. The reporter fluorescence of the beads was determined with a Luminex analyzer and expressed as the median fluorescence intensity (MFI) of at least 100 beads per set per well.

Seropositivity cutoffs for each specific HPV type have been previously defined [12,21]. Briefly, cutoffs were calculated for each type based on the MFI values of serum samples obtained from women presumed to be naïve to HPV infections (371 female students who reported never having engaged in penetrative sexual intercourse and had no evidence of genital HPV DNA for 25 HPV types). The cutoffs were defined as 5 standard deviations above the means of this naïve group. For the present study, a set of 186 informative “bridging” sera, with known antibody status, were tested with our own samples to allow the defined cutoffs to be standardized to the exact assay conditions of the present sample batch. Serology results were dichotomized as antibody positive or negative.

### Statistical methods

For the following analyses, “any HPV” refers to seropositivity or DNA positivity for at least one of the following HR-HPV types: 16, 18, 31, 33, 35, 45, 52, and 58.

Any, type-specific and age-specific (< 20, 21-30, 31-40, 41-50, 51-60, and > =61 years) HR-HPV seroprevalence (at baseline and follow-up), seropersistence, seroclearance and seroconversion (at follow-up) were assessed. Seropersistence was defined as the number of women who were seropositive at baseline and follow-up among all women who were seropositive at baseline. Seroclearance was the number of women who were seropositive at baseline and seronegative at follow-up among all those seropositive at baseline. Seroconversion was the number of women seronegative at baseline and seropositive at follow-up among those seronegative at baseline.

Baseline seropositivity was compared with concurrent cervical DNA positivity to assess concordance, which can only be examined at the type-specific level, as women are often infected with multiple types of HPV and the concordance status can be different for each type. To overcome such limitation, analyses were performed at the genotype level using generalized estimating equation (GEE) models that account for the lack of independence between multiple observations from the same woman [22,23].

Univariable and multivariable GEE models were performed to identify predictors of HR-HPV seroprevalence at baseline. Risk estimates were adjusted by potential risk factors for HPV seropositivity: age at baseline, marital status, education level, number of children, age at first sexual intercourse, lifetime number of sexual partners, high-risk sexual partner (who engaged in extra-relationship sexual activity), history of sexually transmitted disease, hormonal contraception, smoking, condom use and cervical HR-HPV DNA infection. Variables with p-value < = 0.2 in the univariate analysis (age at baseline, age at first intercourse, lifetime number of sexual partners, smoking status, cervical HPV DNA) were then included in multivariate models. Tests for linear trend of ORs were performed by giving an increasing score for each level of the categorized variable and fitting them into the model as continuous variables. Predictors of HR-HPV seropersistence were evaluated by using GEE models, as described above for HPV seroprevalence; no variables had p-value < = 0.2 in the univariate analyses, so estimates presented were adjusted by women’s age at baseline.

All tests were two-sided and the results with p < 0.05 were interpreted as statistically significant. Statistical analyses were performed using STATA version 11.

## Results

The median age of the 1021 women studied at baseline was 43 years (range: 15-86) and for the 600 women at follow-up it was 48 years (range: 19-87). Prevalence of ASCUS+ cytology was 3.7% (37/994) at baseline and 2.8% (16/573) at follow-up. ASCUS+ cytology was five times more prevalent in DNA-positive women (12.8%) than in DNA-negative women (2.6%). On the other hand, ASCUS+ cytology was similarly prevalent in seropositive (3.6%) and seronegative women (3.1%) (data not shown).

Cervical HR-HPV DNA positivity for at least one of the eight studied HR-HPV types was 6.7% at baseline and 8.7% at follow-up. At baseline, the most prevalent types were HPVs 16 (2.5%), 58 (1.3%), 31 (1.1%), 45 (0.7%) and 52 (0.7%), representing 83.3% of DNA-positive women; at follow-up, most prevalent were HPVs 16 (3.0%), 18 (2.5%), 45 (1.7%), 31 (1.0%) and 52 (1.0%), representing 92.3% of DNA-positive women (data not shown).

**Figure 1:**
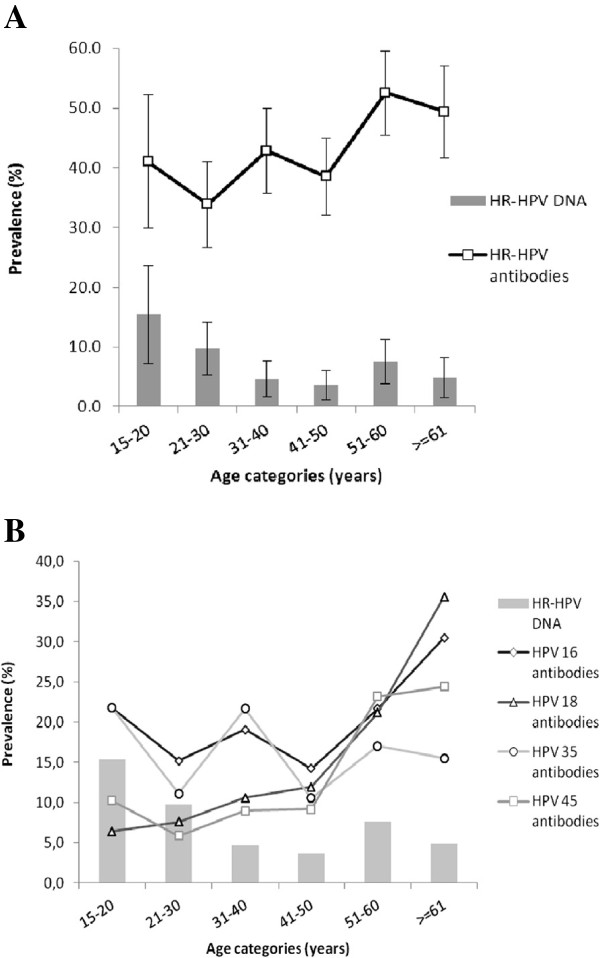
**Age distribution of high-risk human papillomavirus DNA positivity**^**a **^**and (A) any seropositivity**^**a **^**or (B) type-specific seropositivity, in 1021 women from Santiago, Chile, 2001. **^a^Positive for at least one of the following high-risk HPV types: 16, 18, 31, 33, 35, 45, 52, and 58. Lines represent seropositivity and bars represent cervical DNA positivity. HR: high-risk, HPV: human papillomavirus.

At baseline, almost half of the participants were seropositive for at least one of the eight studied HR-HPV types, and seropositivity increased by 16% at follow-up (Table 1). The four most prevalent types were HPVs 16, 18, 35 and 45, representing 88.9% of seropositive women at baseline and 87.4% at follow-up. Nearly half of the seropositive women were so for multiple types at baseline and this proportion increased by 13% at follow-up. An analysis restricted to only the 600 women who were followed also shows a rise in any HPV seropositivity (13% increase, from 44.5% at baseline to 50.2% at follow-up) and in the proportion of seropositive women with multiple types (19% increase, from 42.3% at baseline to 50.5% at follow-up) (data not shown).Figure 1 shows any HPV and type-specific HPV seroprevalence and any HPV DNA prevalence by age among all women who participated at baseline. Any HPV seroprevalence significantly increased with age (p-trend = 0.003); it ranged from 33.9% (age 21-30 years) to 52.6% (age 51-60 years). Although numbers are small, type-specific seroprevalence of the most common high-risk types (HPV 16, 18 and 45), except for HPV 35, followed the same trend. Any HPV DNA prevalence peaked at age 15-20 years (15.4%) declining thereafter until a second, lower peak at age 51 to 60 years (7.6%). Type-specific DNA curves were very unstable due to low number of infections in each age category (data not shown).

**Table 1:** Prevalence of high-risk human papillomavirus antibodies among women from Santiago, Chile, at baseline in 2001 and follow-up in 2006

	**Seropositivity**			
	**Baseline (n = 1021)**		**Follow-up (n = 600)**	
**HPV type**	**n**	**% (95% CI)**	**n**	**% (95% CI)**
Any HR-HPV^a^	442	43.3 (40.2-46.4)	301	50.2 (46.1-54.2)
16	189	18.5 (16.2-21.0)	118	19.7 (16.5-22.9)
18	148	14.5 (12.4-16.8)	110	18.3 (15.2-21.4)
31	92	9.0 (7.3-10.9)	59	9.8 (7.4-12.2)
33	46	4.5 (3.3-6.0)	31	5.2 (3.4-6.9)
35	160	15.7 (13.5-18.0)	109	18.2 (15.1-21.3)
45	142	13.9 (11.8-16.2)	92	15.3 (12.5-18.3)
52	70	6.9 (5.4-8.6)	43	7.2 (5.1-9.2)
58	63	6.2 (4.8-7.8)	44	7.3 (5.2-9.4)
Single type	244	23.9 (21.3-26.5)	149	24.8 (21.4-29.3)
Multiple types	198	19.4 (17.0-21.8)	152	25.3 (21.8-28.8)

^a^Positive for at least one of the following high risk HPV types: 16, 18, 31, 33, 35, 45, 52, and 58.

HR: high-risk, HPV: human papillomavirus, CI: confidence interval.

At baseline, 539 (53.3%) women were negative for both HPV DNA and antibodies, while 33 (3.3%) women were DNA and antibody positive. Seropositivity was 50.0% among DNA-positive women and 57.2% among DNA-negative women (Additional file 2). Table 2 shows the type-specific concordance between HPV DNA and HPV antibody prevalence at baseline, considering each infection in each woman as a unit or count. Seropositivity for each type tended to be higher among DNA-positive infections with the same type, reaching statistical significance only when all infections were analyzed together. In summary, 17 type-specific infections coexisted with their corresponding antibody, while 103 infections did not.

**Table 2:** **Any**^**a **^**and type-specific concordance**^**b **^**between high-risk human papillomavirus cervical DNA positivity and seropositivity, Santiago, Chile, 2001**

**HPV DNA infection**	**HPV seronegative n (%)**	**HPV seropositive n (%)**	**OR of seropositivity for corresponding type (95% CI)**
**At woman level (n = 95)**			
HR-HPV other than HPV 16	51 (72.9)	19 (27.1)	
HPV 16	17 (68.0)	8 (32.0)	1.3 (0.5-3.4)
HR-HPV other than HPV 18	74 (83.1)	15 (16.9)	
HPV 18	5 (83.3)	1 (16.7)	1.0 (0.1-9.1)
HR-HPV other than HPV 31	73 (86.9)	11 (13.1)	
HPV 31	8 (72.7)	3 (27.3)	2.5 (0.6-10.8)
HR-HPV other than HPV 33	84 (91.3)	8 (8.7)	
HPV 33	2 (66.7)	1 (33.3)	5.3 (0.4-64.4)
HR-HPV other than HPV 35	74 (80.4)	18 (19.6)	
HPV 35	1 (33.3)	2 (66.7)	8.2 (0.7-95.8)
HR-HPV other than HPV 45	71 (80.7)	17 (19.3)	
HPV 45	6 (85.7)	1 (14.3)	0.7 (0.1-6.2)
HR-HPV other than HPV 52	76 (86.4)	12 (13.6)	
HPV 52	7 (100)	0	0
HR-HPV other than HPV 58	79 (96.3)	3 (3.7)	
HPV 58	12 (92.3)	1 (7.7)	2.2 (0.2-22.9)
**At infection level (n = 760)**			
Positive to different HR-HPV	582 (85.0)	103 (15.0)	
Positive to same HR-HPV	58 (77.3)	17 (22.7)	**1.7 (1.1-2.5)**

^a^Any HPV refers to HPV types 16, 18, 31, 33, 35, 45, 52, and 58.

^b^Only among HPV DNA positive infections.

Bold: statistically significant results (p-value < 0.05).

HR: high-risk, HPV: human papillomavirus, OR: odds ratio, CI: confidence interval.

Determinants of any HPV seropositivity at baseline are described in Table 3. Seropositivity significantly increased with older age, younger age at first sexual intercourse and greater lifetime number of sexual partners. HPV DNA positivity was also associated with seropositivity, although not reaching statistical significance. No association was observed with smoking status.

**Table 3:** **Determinants of seropositivity against high-risk human papillomavirus**^**a **^**among 1021 women from Santiago, Chile, 2001**

**Characteristics**	**OR**^**b **^**(95% CI)**
**Age (years)**	
15-20	1.00
21-30	0.70 (0.40-1.22)
31-40	1.24 (0.73-2.11)
41-50	1.09 (0.59-1.69)
51-60	**2.15 (1.15-3.32)**
≥ 61	**2.16 (1.17-3.47)**
*p-trend*	***< 0.001***
**Age at first sexual intercourse (years)**	
15	1.00
16-17	0.79 (0.57-1.08)
18-19	**0.66 (0.47-0.92)**
≥ 20	**0.53 (0.38-0.74)**
*p-trend*	***< 0.001***
**Lifetime number of sexual partners**	
1	1.00
≥2	**1.30 (1.01-1.67)**
**Smoking status**	
Never	1.00
Former	0.77 (0.59-1.00)
Current	0.71 (0.36-1.40)
**Cervical HPV DNA**	
Negative	1.00
Positive^c^	1.48 (0.86-2.56)

^a^Seropositivity for at least one of the following high risk HPV types: 16, 18, 31, 33, 35, 45, 52, and 58.

^b^GEE estimates, odds ratios (95% confidence interval) adjusted by all variables included in the table (variables with p-value <= 0.2 in univariate analysis).

^c^Positive for at least one of the following high risk HPV types: 16, 18, 31, 33, 35, 45, 52, and 58.

Bold: statistically significant (p-value < 0.05).

HPV: human papillomavirus, OR: odds ratio, CI: confidence interval.

**Figure 2:**
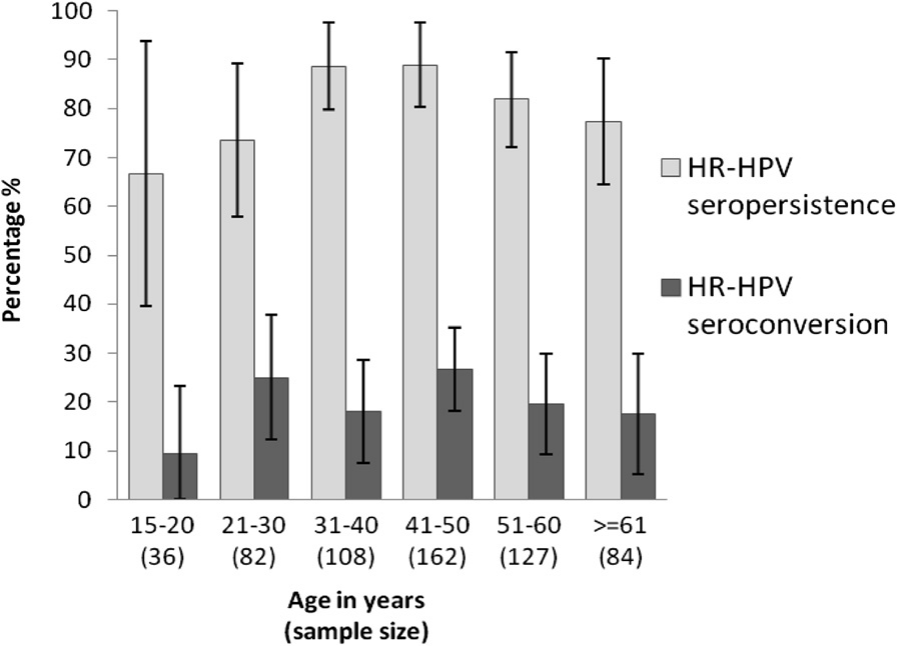
**Age-specific seroconversion and seropersistence of high-risk human papillomavirus antibodies among 600 Chilean women who participated in two cross-sectional visits (2001 and 2006).** HPV types included: 16, 18, 31, 33, 35, 45, 52 and 58. Age categories based on age at baseline. HR: high-risk, HPV: human papillomavirus.

Among the 600 women who participated in both study visits, 43.5% were seronegative at both visits, 8.0% (18.0% of seropositive at baseline) presented seroclearance, 12.0% (21.6% of seronegative at baseline) seroconverted, and 36.5% (82.0% of seropositive at baseline) presented seropersistence. HPV 16 seropersistence and seroconversion were 80.5% (95% CI: 72.8-82.3) and 7.4% (95% CI: 4.8-9.2), respectively (data not shown). Our dataset was under-powered to detect significant differences in seropersistence and seroconversion by age, though seropersistence was high (74-89%) in all age groups, except for ages 15-20 years (which had a very small sample size), and seroconversion was between 18% and 27%, also excluding the youngest age group (Figure 2). There were 16 women with incident DNA infections among whom eight were seronegative at both visits, one presented seroclearance, five presented seropersistence and two presented seroconversion (data not shown). Determinants of seropersistence of any HPV were evaluated and no significant associations were identified (Additional file 3).

## Discussion

In this population-based sample of women aged 15 years and older, from Santiago, Chile, we found a high seroprevalence (43%) of the eight most common HR-HPV types; 41% of women already had antibodies at age 15-19 years. This supports the recommendations that HPV vaccination should begin at younger ages. Interestingly, any HPV and type-specific seroprevalence increased with age even at older ages, consistent with data from cross-sectional studies in Taiwan [24], China [25], Mongolia [21] and England [26]. However, the observed seroprevalence age-trend in Chile differs from reports in the US [27,28], Costa Rica [11], The Netherlands [29] and Australia [30], where seroprevalence peaked one or two decades after sexual debut and steadily declined after ages 50 or 60 years. The declining seroprevalence at older ages is thought to be the result of the waning of antibodies with time after HPV exposure [11]. In our Chilean population, study participants maintained exposure to HPV infection at older ages, as shown by the second peak of DNA infection (53%) around the age of 50-60 years; this exposure could act as a booster, maintaining high antibody titers after age 61 years. A second HPV DNA prevalence peak has been reported as well in other populations; however, it is not clear whether this is the result of incident infections due to new sexual partners, the reactivation of a latent infection associated to immunological changes, or the result of a subgroup of the population that had a higher exposure to HPV in their lifetime (cohort effect) [31].

Life-time number of sexual partners, marital status, HSV-2 infection, oral contraception, smoking and viral load have been postulated in several studies as determinants of HPV seropositivity [11,12,27,32,33]. Our analyses indicate that women who at baseline reported early onset of sexual intercourse and two or more sexual partners in their lifetime had increased odds of being seropositive; this suggests that those women were more exposed to repeated infections, thus had higher antigenic stimulation to develop viral antibodies. Although number of sexual partners was associated with high seroprevalence, women in our study reported having very few lifetime sexual partners (384 and 59 women reported 2 and 3-4 partners, respectively) which suggests that continuous exposure to HPV infection may occur mostly through their current partner. Contrary to several previous studies [12,21,27,33], we did not observe an association of seropositivity with marital status or high-risk sexual behavior of the partner; we cannot rule out that the lack of association between HPV antibodies and high-risk partner could be due to underreporting of this situation. Any HPV and type-specific concordance between seropositivity and DNA positivity was moderate, as has been reported by others [11,12,28] and could reflect that only a subset of women exposed to HPV will seroconvert. Baseline DNA prevalence was significantly associated with baseline seroprevalence (for any HR-HPV). However, it should be noted that our concordance analysis is susceptible to being affected by cross-reactivity of the assay since we were unable to control for this factor.

Data on the natural history of HPV antibody response is limited; few studies report longitudinal measurements of both DNA and antibodies. In our study, the majority of the seropositive women at baseline were also seropositive five years later (82% for any HPV). Previous cohort studies have shown that most women who seroconverted remained seropositive until the end of follow-up (follow-up periods from 18 months to 5 years) [34-36]. Observed HPV 16 seropersistence in our study (80%) was much higher than that reported in a large Costa Rican six-year follow-up study (55%) of women with a similar age distribution [37]; in both studies, seropersistence was constant across different ages. A much lower seropersistence rate (31%) was observed in the US after a minimum follow-up of one year [38]; however this study included only young women (mean age 20 ± 3 years). Regarding seroconversion, we found a relatively high rate (21.6% for any HPV, 7% for HPV 16) with a constant pattern across age groups. Our estimate for HPV 16 seroconversion is similar to the one reported (6%) for Costa Rican women [37]. We must note that since our study included only two assessments five years apart, our estimates of seropersistence and seroconversion, although informative, could be affected by changes that may have occurred during that interval; for example, it is possible that women we identified as seropersistent in fact serocleared and later acquired a new infection in the five-year period.

Limitations of the present study include the referred lack of intermediate assessments and the relatively low sample size that prevented us from performing more informative type-specific analyses. Strengths of the study include the population-based study design and prospective analysis, and the use of a high-throughput serologic test that allows the simultaneous identification of antibodies against eight HPV types.

## Conclusions

This study provides for the first time estimates of seroprevalence, seropersistence and seroconversion of eight HR-HPV types in Chile. These findings are useful to better understand the epidemiology of the immune response to HPV and also to inform cervical cancer prevention strategies in middle developing countries in Latin-America and elsewhere. The high HPV seroprevalence, already elevated below age 20 years, as well as the increase after age 50 years support the initiation of vaccination programs at young ages and the established screening practice at older ages in Chile. This data constitutes a baseline assessment before the incorporation of HPV technologies in cervical cancer prevention.

## Abbreviations

HPV: Human papillomavirus; HR: High-risk; MFI: Median fluorescence intensity; GEE: Generalized estimating equation; OR: Odds ratio; CI: Confidence interval; ASCUS+: Atypical squamous cells of undetermined significance or worse cytology.

## Competing interests

The authors declare that there is no competing interests.

## Authors’ contributions

CF was responsible for the study conception, design and implementation, and analysis and interpretation of data. KP coordinated the field work. PS was responsible for the DNA testing and collaborated with data analysis. MP was responsible for the serological testing and collaborated with data analysis. AD performed the statistical analyses. FC, SF, and VV were responsible for data analysis and interpretation. CF, FC, VV and AD were responsible for the manuscript preparation. All authors revised the manuscript critically and approved the final version for publication.

## Pre-publication history

The pre-publication history for this paper can be accessed here:

http://www.biomedcentral.com/1471-2334/14/361/prepub

## Supplementary Material

Additional file 1: Table S1 Baseline characteristics of women who participated only at baseline and those who participated at both baseline and follow-up. Santiago, Chile, 2001 and 2006. Click here for file

Additional file 2: Table S2 Baseline characteristics of women by serological status. Santiago, Chile, 2001. Click here for file

Additional file 3: Table S3 Determinants for seropersistence of any^a^ high-risk human papillomavirus among women in Santiago, Chile, 2001-2006. Click here for file
